# Structured hernia surgery training program for general practitioners in Rwanda - feasibility and evaluation

**DOI:** 10.1007/s10029-025-03260-8

**Published:** 2025-01-23

**Authors:** Ralph Lorenz, Lea Hubert, Christoph Paasch, Joachim Conze, Chris Oppong, Jacob A. Akoh, David M. Sedgwick, Venuste Nsabimana, René Mantke

**Affiliations:** 1 3+CHIRURGEN Hernia Center, Klosterstrasse 34/35, 13581 Berlin, Germany; 2 Department of General and Abdominal Surgery, Clinic for General and Abdominal Surgery, University Hospital Brandenburg an der Havel, Hochstrasse 29, 14770 Brandenburg an der Havel, Germany; 3 UM Hernienzentrum Dr. Conze, Arabellastraße 17, 81925 München, Germany; 4 Department of General, Visceral, Pediatric and Transplant Surgery, University Hospital Aachen, Aachen, Germany; 5 Nuffield Health Plymouth Hospital, Derriford Road, Plymouth PL6 8BG, UK; 6 Fort William, Inverness-Shire, PH33 7LX, UK; 7 Rwamagama Level II Teaching Hospital, Rwanda, Kigali-Kayonza Rd, Rwamagana, Rwanda; 8 Faculty of Medicine, Brandenburg Medical School Theodor Fontane, Brandenburg, Germany; 9 Faculty of Health Sciences Brandenburg, Brandenburg Medical School Theodor Fontane, Brandenburg an der Havel, Germany

**Keywords:** Hernia training, Surgical education, Self assessment, Competency assessment, Low and middle income countries, General practitioners

## Abstract

**Background:**

Hernias are among the most common surgical conditions worldwide, with significant prevalence in Africa. However, according to recent WHO statistics, Africa faces a critical shortage of trained surgeons. Structured surgical training programs are also scarce. Since 2016, Surgeons for Africa in collaboration with Operation Hernia have developed structured training course on hernia surgery specifically for surgeons in Rwanda. Due to the severe shortage of surgeons, a new initiative was launched in 2023 to train general practitioners (GPs) to support the country’s surgical care needs. This study aims to assess the feasibility and effectiveness of these training programs for general practitioners.

**Methodology:**

Six standardized questionnaires were used to evaluate the structured training program before, during, and after the one-and-a-half-week courses conducted in Rwanda. Both trainees and trainers completed the relevant evaluations. The results were anonymized, ummarized using descriptive statistics, and statistically analysed. Trainers also assessed the surgical competence of each participant at the end of the course.

**Results:**

Between 2023 and 2024, 47 general practitioners received hernia surgery training in several Rwandan hospitals. The course significantly improved both the theoretical knowledge and practical surgical skills of the participants. Of the 47 GPs, 22 were able to independently perform simple inguinal hernia surgeries after the training. Three were able to perform complex inguinal hernia repairs independently. 12 participants required minimal supervision, while 22 required full supervision.

**Conclusion:**

This study confirms the feasibility and effectiveness of a standardized hernia surgery training program for general practitioners in Rwanda. The results demonstrate the rogramme’s potential to address the surgical care gap by enabling GPs to perform basic hernia surgeries.

**Supplementary Information:**

The online version contains supplementary material available at 10.1007/s10029-025-03260-8.

## Introduction

Hernias are among the most common surgical conditions globally, including in Africa. The prevalence of hernias is comparable between Africa and Europe [1]. On average, one in four men and one in 27 women will develop an inguinal hernia in their lifetime [2]. However, the number of emergency surgeries in Africa is significantly higher due to inadequate medical infrastructure and delayed treatment. A severe shortage of qualified staff prevents the safe and effective implementation and execution of operations. According to the Organisation for Economic Co-operation and Development (OECD) Report of 2014, there are 65.4 surgeons per 100,000 inhabitants in Germany, whereas in Rwanda, there are only 0.3 surgeons [3]. This disparity has far-reaching consequences for the health systems in these regions, particularly in emergency surgery and the management of common conditions like hernias. Unlike in Germany, specialized hernia surgery training is not well developed in most African countries. Since 2016, the German aid organization *Surgeons for Africa* in collaboration with the British organization *Operation Hernia* has developed and implemented a structured, specialized hernia surgery training program. Initially designed for surgeons, this program emphasizes training of theoretical and practical skills [4]. Due to the severe shortage of surgeons, a new initiative was launched in 2023 to train general practitioners (GPs) to support the country’s surgical care needs. This study evaluates the feasibility and effectiveness of including general practitioners (GPs) in this specialized hernia training program.

## Methods

A hernia-specific training program [4] developed by *Surgeons for Africa* and *Operation Hernia* was adapted for a new target group: General Practitioners (GPs). In Rwanda, a GP is a relatively junior doctor employed to work in a secondary health facility, performing multiple roles in multiple specialties. The program includes a standardized curriculum for both trainers and trainees, with a two-day theoretical portion and a five-day practical “hands-on” component. Two basic standardized surgical techniques were taught [5]: the Shouldice technique (suture-based) and the Lichtenstein technique (mesh-based).

An evaluation of this specific training program occurred before, during, and after the one-and-a-half-week courses held in 2023 and 2024, using six standardized non-evaluated questionnaires. Developed by British and German hernia experts, these questionnaires included self-assessments of practical competence and theoretical knowledge before and after the course. In addition to self-assessments, trainers also evaluated trainees on these areas after the course, along with overall feedback on the program itself [6, 7].

The study was approved by the Medical University Brandenburg an der Havel on January 29th, 2024, in terms of data protection, with ethics approval under file number 170,012,024-ANF.

### Study objectives

- Can GPs be trained to assist in the surgical management of abdominal wall hernias, alleviating the surgeon shortage?
- Is the limited time frame and training structure sufficient to provide standardized hernia surgery training for non-surgeons in Rwanda, a country with limited medical resources?

### Statistics

Only fully answered questionnaires were included in the analysis. Data was analysed quantitatively and qualitatively, with summary statistics compiled in Excel. No univariate and multivariate analysis took place.

### Study population

As a pilot project, no sample size calculation took place. GPs who were trained in 2023 and 2024 were questioned during the survey at hand.

### Definitions

To classify the hernias the European Hernia Society (EHS) Classification was used. Accordingly small hernias were M1, M2, L1, L 2 hernias. Large primary hernias (European Hernia Classification L3 and M3) scrotal and recurrent hernias (European Hernia Classification R X) were considered as more complex hernias.

An expert hernia surgeon in this context is a surgeon who has performed at least 100 hernia surgeries per year for at least 5 years.

## Results

A total of 47 GPs participated in the structured training program in 2023 and 2024. In March 2023, 28 GPs were trained by British (5) and German (9) hernia experts, followed by 19 GPs in March 2024. Of the 282 questionnaires (6 per participant) received, only fully answered ones were included into the analysis.

Participants reported a wide range of prior surgical experience: an average of 89 hernia operations, 34 laparotomies, 728 minor surgeries, and 979 caesarean sections per participant (Fig. Supplement 1). The theoretical part of the course in 2023 received high ratings, with 15 out of 17 categories scoring above 4 out of 5, indicating a “very good” quality. The remaining two categories were scored as “good” (Table 1).

**Table 1:** Evaluation of the lecturing trainers by the trainees 2023 rating in school grade system: (weak = 1, average = 2, good = 3, very good = 4, excellent = 5), (*n* = 28)

Kategory	Average rating
Anatomy	4.12
Diagnose/ Differential-Diagnose	4.38
Indication for surgery	4.2
Mesh implantation technique	4.2
Pure Tissue Technique without mesh	4.08
Mesh implantation: principles	4.08
Video LICHTENSTEIN technique	4.31
Pure Tissue Technique without mesh: principles	4.04
Video SHOULDICE technique	4.16
Technique of local anesthesia	4.08
Interactive session: Scrotal hernias	4.04
Pediatric Hernia Care	3.47
Femoral -Hernias	3.87
Complications and their prevention	4.33
Ventral Hernias	4.33
Interactive session: Incarcerated hernias	4.66
Outcome by means of remote monitoring	4.13

**Fig. 1:**
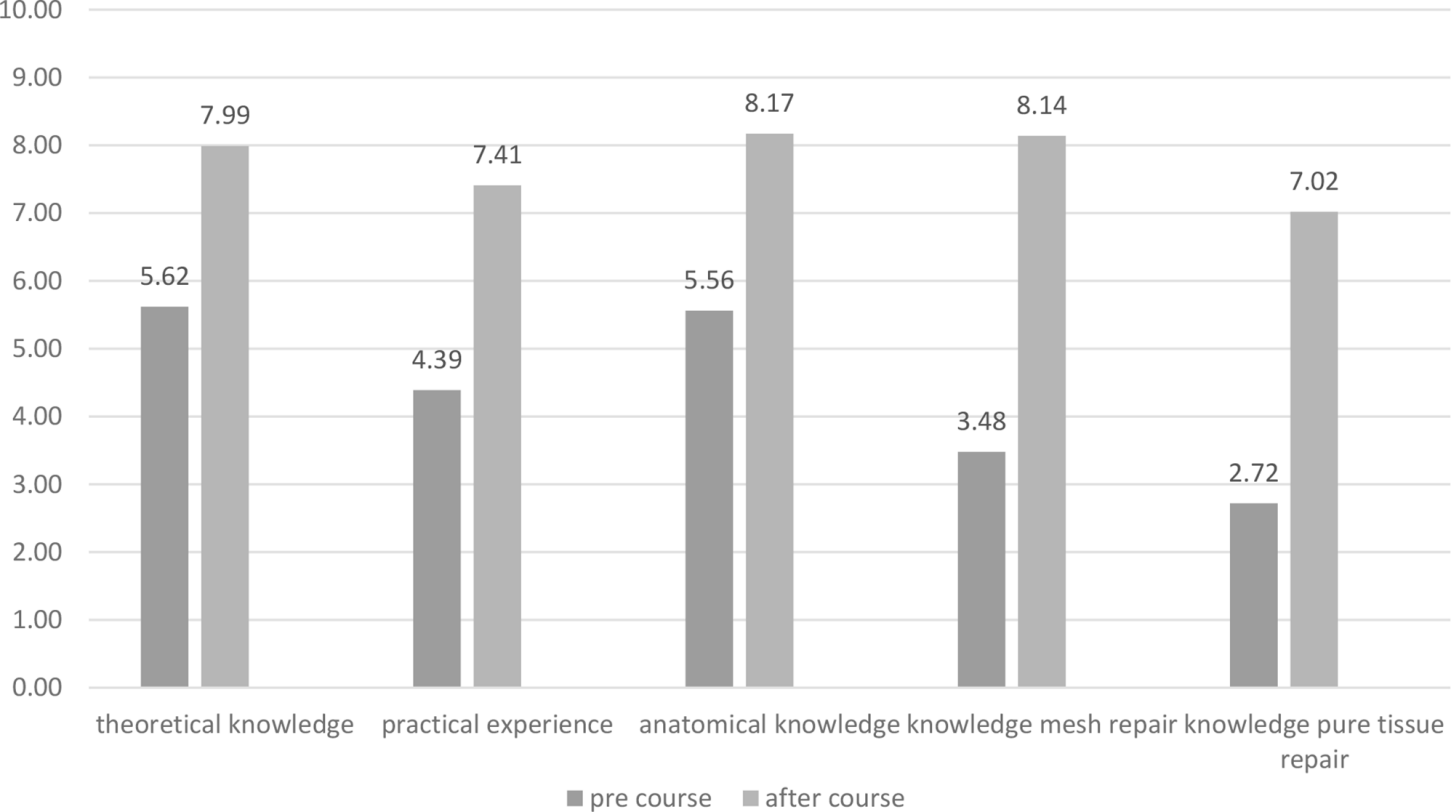
Self-assessment of the trainees’ average theoretical knowledge before and after the training programme in school grades on a scale of 1–10 (1 = poor, 10 = excellent), (*n* = 47)

Theoretical knowledge improved significantly across all areas. Before the course, trainees rated (1 = poor, 10 = excellent) their theoretical knowledge at 5.62 on average; this increased to 7.99 after the course (*n* = 47). Similarly, surgical skills were rated at 4.39 before and 7.41 after the course (*n* = 47). Anatomy knowledge rose from 5.56 to 8.17 (*n* = 47), while competency in the Lichtenstein technique improved from 3.48 to 8.14, and the Shouldice technique from 2.72 to 7.02 (*n* = 47) (Fig. 1).

**Fig. 2:**
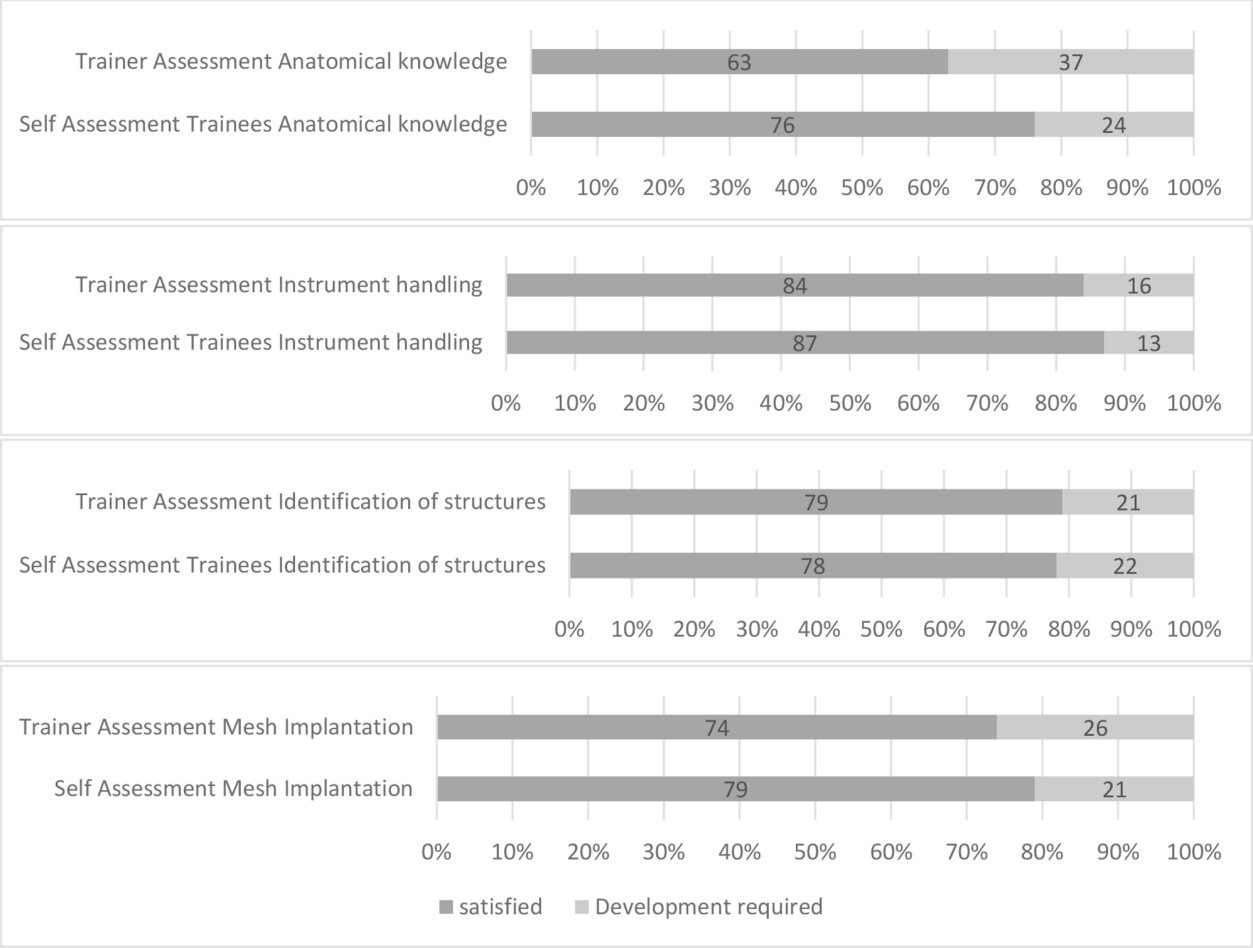
Assessment of practical competence in percent (%) after the training program: self-assessment of trainees vs. assessment by trainers 2023/2024 (*n* = 47)

After the course, trainees’ self-assessments were compared with trainer evaluations. In most categories, self-assessments were more positive than trainer evaluations. However, in the “Implantation of hernia meshes” category, trainers rated trainees slightly higher than the trainees themselves. Notably, the largest difference between self-assessments and trainer evaluations occurred in anatomical knowledge, where trainees rated themselves more favourably (Fig. 2).

**Fig. 3:**
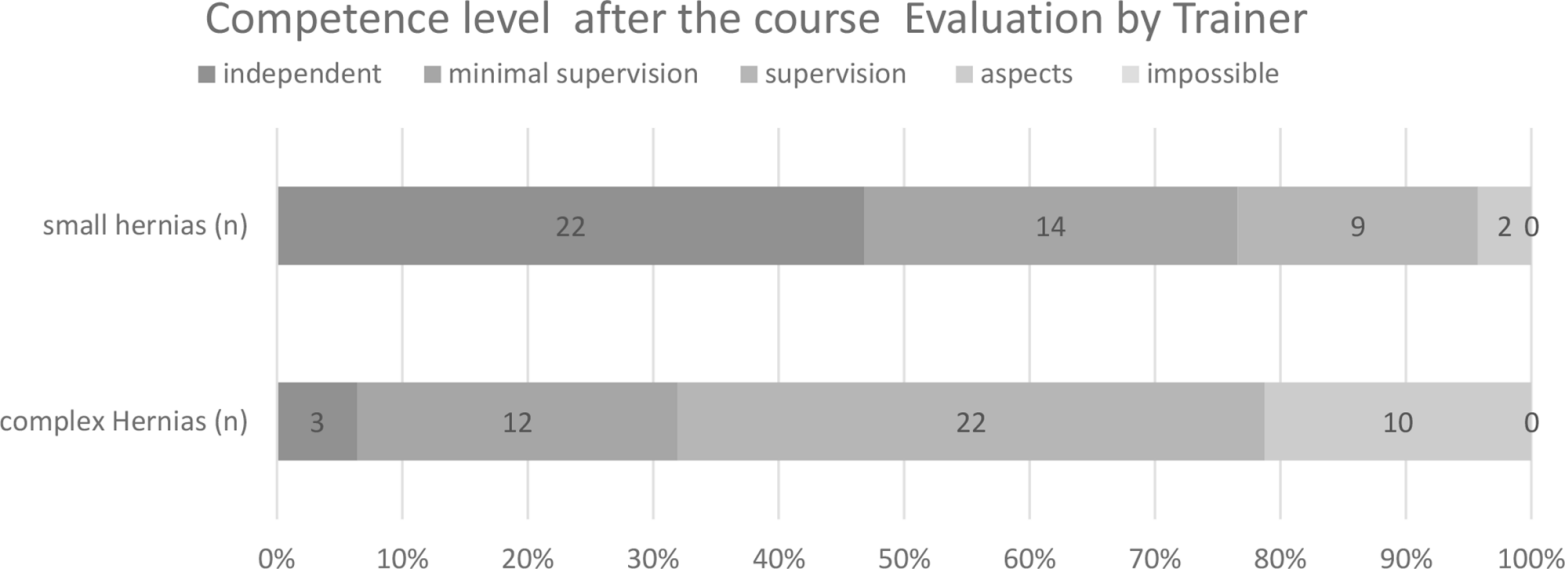
Trainers’ assessment of trainees regarding their ability to perform minor and more complex procedures after completing the training program in numbers and percent, 2023/2024 (*n* = 47)

Post-course assessments showed that 22 out of 47 GPs (*n* = 47) (46,8%) were capable of performing simple inguinal hernia operations independently, whereas only 3 (6,4%) were able to perform more complex hernia surgeries without supervision. Most trainees (34 out of 47) (72,3%) required any supervision for these more complex procedures (Fig. 3).

## Discussion

According to the *Lancet* Commission’s report, there is a notable discrepancy in access to surgical care and the availability of mesh for hernia repairs between low- and middle-income countries (LMICs) and high-income countries (HICs) [8]. In low-income countries (LICs), patients are more likely to present with complicated cases, such as incarcerated or strangulated hernias, due to delayed access to care [9]. The lack of mesh in LMICs has resulted in more traditional suture repairs which results in higher recurrence rates and inferior long-term outcomes for patients [9].

Abass and co-workers highlight the severe shortage of qualified surgical staff as a major barrier to safe and effective surgical care, especially in LMICs [10]. For example, Rwanda has only 0.3 surgeons per 100,000 inhabitants, compared to 65.4 surgeons in Germany [3]. This shortage exacerbates the healthcare crisis, particularly in Africa and Southeast Asia, where there is a significant gap in access to surgical, anaesthetic, and obstetric services.

Financial barriers also heavily influence patient access to surgical care. In Sierra Leone, for example, 27 out of 39 patients refused necessary hernia treatment due to financial constraints [11]. The situation is similar across many LMICs, where emergency surgeries are more common, with higher risks of complications and fatalities compared to industrialized nations. Despite the similar incidence rates of hernias between African countries and Europe, emergency hernia surgeries are disproportionately higher in Africa due to delayed medical interventions [12].

Studies in other countries such as Ghana and Uganda also indicate significant obstacles to hernia treatment [13–16]. In Ghana, for instance, of 530,082 patients needing surgical treatment for inguinal hernias, only a small proportion received care due to financial difficulties, misconceptions about the severity of the condition, and long delays in seeking medical help [17]. A similar study in Uganda found that about 50% of male patients did not experience pain, further complicating the urgency for surgery [15]. These findings underline the multifactorial causes of delayed surgical treatment in LMICs, which includes financial constraints, lack of awareness, and inadequate medical infrastructure.

Despite these challenges, there are promising developments. Standardized training programs in LMICs have shown success in addressing surgical skills gaps, particularly in hernia surgery. For example, a program in São Paulo, Brazil, has laid the foundation for sustainable education in underserved populations [18].

Surgical task shifting and task sharing, where non-surgeon clinicians (NSCs) are trained to perform specific surgical procedures, have been proposed as a potential solution to addressing the shortage of surgical workforce in low-resource settings [19, 20]. A comparative randomized study from Sierra Leone demonstrated the equivalence of elective hernia surgery performed by Associate Clinicians vs. Medical Doctors [21]. A study from Malawi demonstrated the feasibility of training non-medical staff to perform certain surgical procedures with results comparable to those of trained surgeons [22]. Such training models are promising to expand the healthcare workforce, improving access to surgical care in LMICs.

The study sought to explore the feasibility and effectiveness of a time-limited, structured hernia surgery training program for GPs in Rwanda, a low- and middle-income country. In this study, 22 out of 47 trainees were able to carry out uncomplicated non-complex hernia surgeries independently after completing the course, though only 3 of them felt comfortable to perform more complex procedures. This suggests that, while the program shows potential, especially for simpler surgeries, there is a need to enhance training to cover more complex surgical tasks. Another notable factor is the variation in knowledge and practical skill levels among the trainee surgeons at the beginning of the program. This discrepancy, while not entirely controllable, could impact the trainees’ learning outcomes. Also, it must be borne in mind that this structured training in hernia surgery equipped trainees with many generic and transferrable skills applicable to other conditions.

To enhance surgical capacity in Rwanda and other LMICs, long-term follow-up and strategic investment in training programs are needed. The results of this study clearly indicate that the one-and-a-half-week hernia surgery training program for general practitioners in Rwanda led to substantial improvements in both theoretical knowledge and practical surgical skills. The success of the program was shown through quantitative and qualitative evaluations, demonstrating its effectiveness in addressing the shortage of surgical professionals in a low- and middle-income country like Rwanda. Overall, the program has proven to be a practical and beneficial approach to expanding surgical capacity in Rwanda, though it also calls for strategic enhancements, such as extended training durations, further advanced courses, and continuous evaluation, to ensure long-term impact and sustainability. Further structured education programs have the potential to significantly improve the surgical landscape in LMICs.

In addition, trainees are provided with assistance via video calls to address possible complications. In addition, discussions are underway to conduct training with trainees who have already attended training courses. Since 2022, the two charity organizations have developed and conducted the first train-the-trainer courses in 2022 in order to establish contact persons for more complex hernia cases in Rwanda as well. This could ensure long-term impact and sustainability.

## Limitations

The study has several limitations. Firstly, the small number of trainees coupled with the fact that not all participants completed the questionnaires correctly or consistently restricts the ability to make broad or definitive claims about the program’s benefits. This highlights the need for further studies to improve the reliability and generalizability of the findings. Secondly, the short duration of the program, which is conducted annually, restricts the ability to fully assess the long-term impact of the training on skill retention and practical application. As such, repeated studies and potential adjustments to the observational methodology are necessary to enhance interpretability. Thirdly, although the trainers aimed for objectivity in their assessments, a degree of subjectivity is unavoidable, which may have influenced both the training outcomes and the overall significance of the study.

In summary, while the program has shown promising results, the limitations in sample size, study duration, and assessment variability necessitate further investigation to obtain more definitive conclusions about the long-term benefits and effectiveness of the training initiative.

## Conclusion

This study demonstrates the feasibility of a structured training program in hernia surgery for GPs in Rwanda, with significant improvements in both theoretical and practical skills. To enhance surgical capacity in Rwanda and other LMICs, long-term follow-up and strategic investment in training programs are needed. The results of this study clearly indicate that the one-and-a-half-week hernia surgery training program for general practitioners in Rwanda led to substantial improvements in both theoretical knowledge and practical surgical skills. The success of the program was shown through quantitative and qualitative evaluations, demonstrating its effectiveness in addressing the shortage of surgical manpower in LMICs like Rwanda. In summary, while the program has shown promising results, the limitations in sample size, study duration, and assessment variability necessitate further investigation to obtain more definitive conclusions about the long-term benefits and effectiveness of the training initiative.

## Electronic supplementary material

Below is the link to the electronic supplementary material.

Supplementary Material 1

Supplementary Material 2

## Data Availability

Data are available on request.
