# Supplementary material for: Structured hernia surgery training program for general practitioners in Rwanda - feasibility and evaluation
Source: Hernia. 2025 Jan 23;29(1):73. doi: 10.1007/s10029-025-03260-8 (PMC11759471; doi:10.1007/s10029-025-03260-8)
Supplement: Supplementary file 1 — Supplementary Material 1 [file 10029_2025_3260_MOESM1_ESM.docx]

*Figure Supplement 1: Overview of trainees' previous surgical experience differentiated by average surgical procedures in total numbers performed independently in 2023/2024 (n=47)*
